# The Potential Use of Artificial Intelligence in Irritable Bowel Syndrome Management

**DOI:** 10.3390/diagnostics13213336

**Published:** 2023-10-29

**Authors:** Radu Alexandru Vulpoi, Mihaela Luca, Adrian Ciobanu, Andrei Olteanu, Oana Bărboi, Diana-Elena Iov, Loredana Nichita, Irina Ciortescu, Cristina Cijevschi Prelipcean, Gabriela Ștefănescu, Cătălina Mihai, Vasile Liviu Drug

**Affiliations:** 1 Faculty of Medicine, Department of Internal Medicine, University of Medicine and Pharmacy “Grigore T. Popa”, 700111 Iasi, Romania; vulpoi.radu@yahoo.ro (R.A.V.); olteanuandrei@yahoo.com (A.O.); iovdiana95@gmail.com (D.-E.I.); lory_nichita@yahoo.com (L.N.); irinaciortescu@yahoo.com (I.C.); cristinacijevschi@yahoo.com (C.C.P.); gabriela.stefanescu@gmail.com (G.Ș.); catalinamihai@yahoo.com (C.M.); vasidrug@email.com (V.L.D.); 2 Emergency Clinical Hospital “Saint Spiridon”, Institute of Gastroenterology and Hepatology, 700111 Iasi, Romania; 3 Institute of Computer Science, Romanian Academy-Iasi Branch, 700481 Iasi, Romania; mihaela.luca58@gmail.com (M.L.); adrian.ciobanu@iit.academiaromana-is.ro (A.C.)

**Keywords:** irritable bowel syndrome, artificial intelligence, diagnostic, deep learning

## Abstract

Irritable bowel syndrome (IBS) has a global prevalence of around 4.1% and is associated with a low quality of life and increased healthcare costs. Current guidelines recommend that IBS is diagnosed using the symptom-based Rome IV criteria. Despite this, when patients seek medical attention, they are usually over-investigated. This issue might be resolved by novel technologies in medicine, such as the use of Artificial Intelligence (AI). In this context, this paper aims to review AI applications in IBS. AI in colonoscopy proved to be useful in organic lesion detection and diagnosis and in objectively assessing the quality of the procedure. Only a recently published study talked about the potential of AI-colonoscopy in IBS. AI was also used to study biofilm characteristics in the large bowel and establish a potential relationship with IBS. Moreover, an AI algorithm was developed in order to correlate specific bowel sounds with IBS. In addition to that, AI-based smartphone applications have been developed to facilitate the monitoring of IBS symptoms. From a therapeutic standpoint, an AI system was created to recommend specific diets based on an individual’s microbiota. In conclusion, future IBS diagnosis and treatment may benefit from AI.

## 1. Introduction

Irritable bowel syndrome (IBS) is a common gastrointestinal disorder, which is estimated to affect around 4.1% of the global population [1]. IBS has a negative impact on the quality of life of patients, while at the same time posing a significant burden on healthcare systems worldwide. People suffering from this condition usually experience symptoms such as abdominal pain, discomfort, or bloating, as well as changes in bowel habits and stool consistency [2]. Even though the pathogenesis of IBS is still largely unknown, a variety of potential mechanisms have been proposed, such as dysbiosis, visceral hypersensitivity, intestinal dysmotility and dysregulation of the gut-brain axis [3]. Additional factors, including cultural influences, frequent antibiotic use, food additives that alter the intestinal microbiota, as well as stress, have been suspected [4].

In the clinical setting, establishing a definitive IBS diagnosis can sometimes be rather challenging. IBS is frequently diagnosed following multiple investigations to rule out organic lesions. However, the Rome criteria have provided a basis for a definitive diagnostic process based mainly on symptoms. Currently, the latest Rome IV criteria represent guideline recommendations for diagnosing IBS [5]. The economic burden of IBS is significant. A meta-analysis by Flacco et al. noted that the costs of IBS in European countries with universal healthcare coverage were around €2889/year (95% CI: 2318–3460) per patient, ranging from €1602 (insurance-based health systems) to €3909 (studies adopting a societal perspective) [6].

The lack of a specific biomarker for IBS diagnosis and management represents a major reason behind these costs. Furthermore, persistent symptoms despite numerous treatment strategies, drive the patient towards more invasive additional investigations, such as colonoscopy. Despite the recommendation of both the American College of Gastroenterology (ACG) [7] and the British Society of Gastroenterology (BSG) [8] against the routine use of colonoscopy in IBS patients without “alarm symptoms”, a national survey in the US found that one-quarter of all colonoscopies were performed on patients with symptoms suggestive of IBS [9].

Undoubtedly, IBS is one of the most difficult conditions to diagnose and manage; however, with the rapid development of technology in medicine, there is an increasing potential for novel tools and strategies to diagnose and treat this disease.

## 2. The Development of Artificial Intelligence in the Medical Field

Artificial Intelligence (AI) was first defined in 1950 by Alan Turing in his work entitled “Computing Machinery and Intelligence”. He stated that “Artificial intelligence is the science and engineering of making intelligent machines, especially intelligent computer programs” [10]. Omnipresent now, the name of this domain was further consolidated in 1956 during the Dartmouth Summer Research Project on Artificial Intelligence. In computer science, AI is a way of modeling a computer system to behave like a human. The ability of AI to acquire, adapt and apply knowledge has had a significant impact on a number of fields, including medicine [11]. The well-known MYCIN study is regarded as the first attempt to automate medical diagnosis and therapeutic recommendations. A backward chaining expert system that involved AI was used to identify bacteria and recommend antibiotic treatment [12].

Many classic methods were used in the early days of AI, such as rule-based systems, neural networks, statistical methods, signal, image and video processing. Some of them used fuzzy, probability, possibility, and chaos theories. The majority of these were conducted offline as a result of time-consuming computations. In the last decade, the development of parallel computing and multi-core graphics processing devices (GPUs) has paved the way for the diversification of machine learning and deep learning structures to take a giant leap forward [13,14].

Recently, AI technology has made significant progress, allowing the use of real-time tools to provide assistance in a variety of medical procedures. Additionally, AI technology is now capable of performing a wide range of tasks. These include assisting the decision-making process in diagnosis and therapy, reducing medical errors, improving productivity, stratifying diseases and predicting risks [15,16].

The potential role of AI in the clinical setting may be immense. Fundamentally, there are three levels on which AI could be implemented in the clinical context.

Firstly, AI can work as a screening tool by identifying individuals who might benefit from a referral for an in-person examination. This might theoretically reduce the burden on healthcare systems [15].

The second role that AI can fulfill is performing an activity previously carried out by humans. Although fully replacing healthcare providers with machines is quite unlikely to ever be possible, certain repetitive time- and resource-consuming tasks can be automated and performed by a well-trained AI system [15].

The third and possibly the most significant role of AI in the medical field is to augment the abilities of human healthcare providers, thus enabling them to maximize the effectiveness of their care. It has been shown that when clinicians and AI work together, the results are significantly better than when they work separately [15,17].

## 3. IBS and Artificial Intelligence

**Figure 1:**
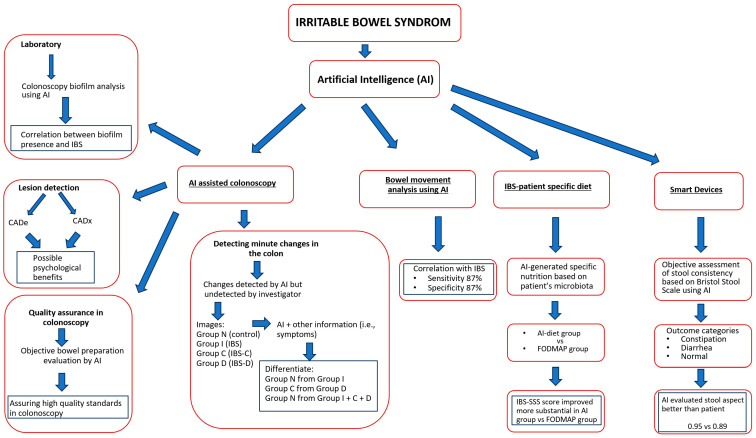
Artificial intelligence in IBS. Abbreviations: AI, Artificial Intelligence; IBS, Irritable Bowel Syndrome; IBS-C, Irritable Bowel Syndrome with Constipation; IBS-D, Irritable Bowel Syndrome with Diarrhea; CADe, computer aided detection; CADx, computer aided diagnosis; FODMAP, fermentable oligosaccharides, disaccharides, monosaccharides and polyols; IBS-SSS, Irritable Bowel Syndrome Severity Scoring System.

Even though the Rome IV symptom-based criteria are the current gold standard for diagnosing IBS, many physicians still perform invasive testing to rule out organic lesions before confirming this diagnosis. This is mainly because of the similarity in clinical presentation between IBS and other organic diseases such as Crohn’s disease, ulcerative colitis and celiac disease [18]. Only a minority of IBS patients are found to have intestinal inflammation during colonoscopy. Despite this fact, primary care physicians tend to refer patients for colonoscopy and biopsy to confirm the diagnosis [19]. Novel technologies such as AI and machine learning can be used to assist in diagnosing IBS, as these can identify certain patterns in medical data that would otherwise be missed. AI could also aid in reducing the number of unnecessary referrals for colonoscopy and biopsy, thus saving time and resources. Throughout the past few years, this technology has gained increasing popularity in a wide range of medical fields, including neurogastroenterology (Figure 1).

### 3.1. Artificial Intelligence-Assisted Colonoscopy in IBS

Colonoscopy is considered the gold standard for diagnosing organic lesions of the colon. Direct visualization of the lesion and the ability to take biopsies represent the two greatest advantages of this investigation. Colorectal cancer (CCR) remains a major public health problem, responsible for over 900,000 deaths worldwide in 2020. According to GLOBOCAN 2020, it represents the second most common cause of cancer-related death [20]. Considering these data, clinicians may have a low threshold of suspicion in patients with chronic abdominal pain and altered bowel movements. In clinical practice, colonoscopy is frequently used in order to rule out organic lesions in patients with symptoms suggestive of IBS. This is in stark contrast with the recommendations of the ACG and BSG on IBS management [7,8]. According to these guidelines, colonoscopy should be reserved solely for patients presenting with alarm signs such as iron deficiency anemia, rectal bleeding or melena, nocturnal diarrhea, unintentional weight loss, symptom onset at an older age (e.g., age ≥ 45 or 50 years), family history of CRC and inflammatory bowel diseases [21].

For a positive diagnosis of IBS, conventional colonoscopy has not proved to be of significant benefit. The results of a recent meta-analysis demonstrated that there is no difference in the yield of CRC or inflammatory bowel disease between individuals with or without IBS [21].

Recently, Tabata K et al. published a paper in which they used a free AI algorithm created with “Google Cloud Platform AutoML Vision” [22]. Their aim was to establish if the AI system is capable of detecting minute changes in the colon that cannot be detected by human investigators. This is usually possible by adding additional information in the training program such as the presence or absence of symptoms. In the study 4 different groups were created: Group N with healthy volunteers, Group I with patients with IBS, Group C patients with constipation-IBS and Group D for patients with diarrhea-IBS. A total of 2479, 382, 538 and 484 images from colonoscopy were randomly selected for groups N, I, C and D, respectively group for training, validation and testing of the AI System. The algorithm managed to discriminate between Group N and Group I with a total area under the curve (AUC) of 0.95 (Group I AUC 0.48, Group N AUC 0.97) and the sensitivity, specificity, positive prediction value and negative prediction value of Group I detection were 30.8%, 97.6%, 66.7% and 90.23%, respectively. Similar but slightly less accurate results were obtained when comparing Group C and D, and Group N vs. Group I + Group C + Group D. Although the experiment shows promising results, the authors themselves recognized that the way the AI classifier distinguishes between endoscopic features from model patients with IBS vs. healthy control is unclear. The problem with these results is also illustrated by the low sensitivity obtained (38%). When predicting whether a patient has IBS or not, it is important that the sensitivity be incredibly high so that as many positive cases as possible can be captured. Until now, no other studies have employed the use of AI technology developed for colonoscopy in order to investigate patients with IBS [22].

**Figure 2:**
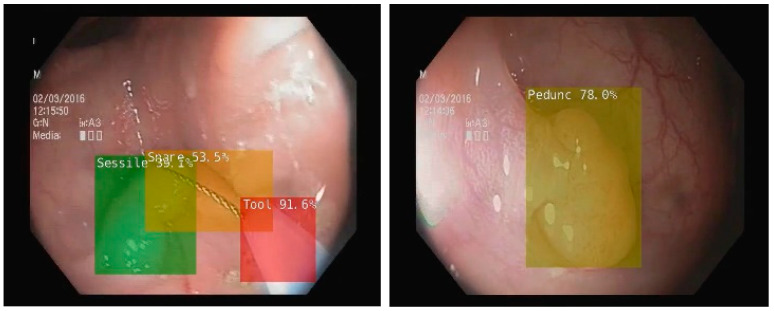
CADe in colonoscopy on NVIDIA Jetson Xavier NX microsystem using MobileNet1 deep learning network retrained to detect more types of polyps, bleedings, water jet, and endoscopic instruments (snare, tool) [25].

To enhance the colonoscopy examination, several other AI systems were developed and approved for use. Nonetheless, most of them were aimed at detecting and diagnosing organic lesions (Table 1) [13,23]. To achieve this, two concepts were introduced in the development of AI-enhanced colonoscopy: computer-assisted detection (CADe) and computer-assisted diagnosis (CADx). Using CADe, AI is able to assist endoscopists in colon lesion detection, thereby increasing the adenoma detection rate (ADR). We present an example from our work of developing an AI system including a CADe module, in Figure 2 [24,25]. For this system, we used MobileNet1, a deep learning network with 4.2 million parameters already trained on the ImageNet dataset, retrained for detecting several types of polyps, lesions, water jet and endoscopic instruments [26]. On the other hand, an AI system using CADx assists the investigator in distinguishing lesions and assessing their potential for malignancy [27]. For this system, the authors used an architecture based on EfficientNet, a neural network with 19 million parameters [28]. This network was also pretrained using the open ImageNet dataset. However, IBS is not associated with any organic lesions. Therefore, it is likely that the benefits of using AI technology in IBS patients will be rather indirect. If no lesions are found during the colonoscopic examination, it may be possible to improve the reliability of an IBS diagnosis by using a tool that objectively analyses the images.

Additionally, AI technologies were developed in order to increase quality assurance in colonoscopy. These AI systems were specifically trained to evaluate quality indicators in colonoscopy such as the rate of cecum intubation and total colonoscopy, withdrawal time, as well as the degree of bowel cleansing according to established bowel preparation scales (e.g., Boston Bowel Preparation Score—BBPS). In the near future, we might be able to ensure that the colonoscopic investigation is of adequate quality by utilizing a tool that can objectively evaluate bowel preparation. In addition to that, AI is associated with a very low risk of missing an adenoma. As a result, both clinicians and patients can rest assured knowing that the IBS diagnosis is indeed accurate, and no further investigation is warranted.

In a paper published by Young Lee et al. [29] an AI system was developed to assess the cleanliness of the bowel during colonoscopy in real-time. The system named ENDOANGEL, used the PyTorch architecture, which is an open-source deep learning framework built to be flexible and modular for research [30]. The study used two trained convolutional neural networks. The first one was designed to determine whether a video frame was appropriate or inappropriate for scoring with the BBPS. The second AI network was developed and trained using a set of appropriate frames annotated with BBPS segment scores (0–3) by experts. To validate the AI system, two expert gastroenterologists were assigned the task of evaluating and assigning a BBPS segment score to a set of colonoscopy video clips. Afterwards, the AI system was given the task of providing a mean BBPS and its performance was compared to human rating. The AI system was able to detect inadequate bowel preparation for each segment with a sensitivity of 100%, while the agreement rates between experts and the system ranged between 68.9% and 89.7%. These findings were consistent with experienced clinicians [29].

Furthermore, artificial intelligence technology has been developed to indirectly assist colonoscopy in a laboratory setting. Baumgartner et al. [4] published a study in 2021 that evaluated the significance of intestinal mucosal biofilms present during colonoscopy. Biofilm formation is a unique form of microbial growth environment in which adherent prokaryotic communities embed themselves within an extracellular matrix. The researchers used an AI system to evaluate the characteristics of biofilms and to study their significance. When the bowel is adequately prepared, biofilms can be observed in white light high-resolution endoscopy as adherent layers on the intestinal surface. These are also resistant to jet washing and detach in a film-like manner [4].

The study, which included 1112 patients from two European medical centers, examined the association between IBS and endoscopically visible biofilms. Out of them, 117 patients (56 with IBS, 25 with ulcerative colitis, and 36 controls undergoing colorectal cancer screening with normal findings at colonoscopy) were selected for molecular and microscopic analyses. Biopsy samples were retrieved from both biofilm-positive and biofilm-negative patients. When no biofilm was present, the biopsies were taken from similar areas of the intestine (cecum or ascending colon) [4]. The AI system used in confocal microscopy employed a trained U-Net, which represents a convolutional network architecture, using skip connections between the encoding and the decoding paths, for fast and precise segmentation of biomedical images. This algorithm was effective in quantifying the number and density of bacteria in biofilm-positive biopsies, as well as confirming the direct contact between the bacteria and the epithelium, which was absent in biofilm-negative biopsies. According to the findings of this study, in 57% of the IBS patients, biofilm was present. Other clinical situations in which biofilm was identified included subjects with ulcerative colitis (34%), after organ transplantation (23%) and healthy individuals undergoing screening colonoscopies (6%) [4].

Briefly, several studies suggest that AI-assisted biofilm analysis could be a potential novel diagnostic tool for IBS.

### 3.2. The Analysis of Acoustic Bowel Movements Using Artificial Intelligence

Over the past few years, there has been a growing interest in research into the sounds of the bowel as a possible non-invasive, reliable, replicable and cost-effective diagnostic test for IBS. The paper published by Craine et al. at the beginning of the twenty-first century was a pivotal study on the topic. In their study, the researchers investigated bowel sounds from both healthy and IBS individuals using computer analysis. According to their findings, fasting sound-to-sound intervals were significantly different between groups. The sensitivity and specificity of this method for detecting IBS were 89% and 100%, respectively [31]. The study of bowel sounds has been undertaken using a variety of analytical approaches such as wavelet transformations, multilayer perceptrons, independent component analysis and autoregressive moving average models. Due to a lack of standardization regarding automated bowel sound analysis, healthcare providers still have a hard time accessing bowel sound devices despite technological advancements in this field [32].

Based on a systematic review of bowel sound analysis methods published in 2018, Inderjeeth et al. concluded that the methods available up to that point were not suitable for evaluating bowel sounds in the clinical setting [33]. A study by the same team, led by Marshall, demonstrated that piezoelectric transducers can be used as contact microphones to identify migrating motor complexes and tested their prototype using machine learning algorithms [18,34]. Their AI system used a logistic regression model based on a calculated IBS Acoustic Index derived from 26 bowel sound features. Using an independent cohort of participants (comprised of recordings from 31 IBS and 37 healthy participants), their proof-of-concept study on bowel sound analysis for diagnosing IBS using AI achieved 87% sensitivity and specificity [18].

Further studies are still needed before automated bowel sound identification procedures can be routinely applied in clinical practice.

### 3.3. Artificial Intelligence-Generated Personalized Diet in IBS

IBS is therapeutically challenging, which is reflected in the patients’ numerous medical appointments. Given the fact that multiple factors are suspected to contribute to disease progression and severity, alongside widely varying symptoms, it is often difficult to design a targeted treatment plan. A specific dietary approach is frequently one of the key components of IBS management, which has been shown to be effective and safe. Certain diets may lead to symptomatic improvement, such as the low fermentable oligosaccharides, disaccharides, monosaccharides, and polyols (FODMAP) diet [35]. Unfortunately, this diet is associated with rather low compliance rates as it is difficult to implement and a fair proportion of IBS patients have no positive response.

Constructing a targeted, specific diet for IBS patients seems a promising strategy. The human microbiome is influenced by ingested foods and may play a significant role in the pathogenicity of the disease. Therefore, it should constitute an essential factor to consider when choosing the right diet for the treatment of IBS. In their work published in Gut Microbes in November 2022, Karakan and his team focused their research on this topic [1]. However, choosing the appropriate diet according to the patient’s microbiome can be rather difficult. In a systematic review of the intestinal microbiota published in 2018, there were many differences and inconsistencies between individuals with and without IBS [36].

Karakan et al. developed an AI system (ENBIOSIS) that may optimize the personalized nutritional strategy based on a patient’s individual microbiota. Their system is a machine learning classifier that uses XGBoost, a stochastic gradient-boosting classification model. For training purposes, two databases were needed. Firstly, they compiled a microbiota database using available open-source data from the American Gut Project [37], the Human Microbiome Project [38] and the Flemish Gut Flora Project [39]. Afterward, they compiled a nutrient database containing various categories, such as carbohydrates, proteins, lipids, vitamins/minerals, phytochemicals, food additives, and specific and fermented foods. For validation purposes, the researchers included two groups of subjects. One was comprised of IBS patients (diagnosed according to the Rome IV criteria), while the other one consisted of healthy subjects. Stool samples were collected from each group at two time points (pre- and post-intervention). High-throughput 16S rRNA sequencing was performed on the fecal samples in order to evaluate the gut microbiota community. Different microbiota patterns were observed in IBS patients compared to healthy controls. In the interventional part of the study, the 34 IBS participants were further divided into two groups. The first group (n = 14) was given a personalized type of microbiota-specific diet, while the second group (n = 11) received a standard IBS diet (low-FODMAP diet). Both groups were followed up for six weeks [1].

The IBS severity scoring system (IBS-SSS) was used to evaluate the clinical status of the participants both pre- and post-intervention. Additionally, changes in the microbiota were followed pre- and post-intervention with the help of a microbiome-derived IBS index score created using a machine learning technique. Even though the microbiota-derived IBS index score and the IBS-SSS improved in both groups (AI-personalized diet vs. low-FODMAP diet), the results were more substantial in subjects receiving an AI-personalized diet [1].

Therefore, it seems that in the future AI is likely to play a significant role in the treatment of a variety of diseases, including difficult-to-treat conditions such as IBS.

### 3.4. Smartphone Application Using Artificial Intelligence to Monitor IBS Symptoms

In today’s world technology is practically ubiquitous, also reflected in the presence of gadgets at every step. Leaving home without a smartphone, a smartwatch, or even smart headphones seems almost inescapable. Nowadays, these gadgets are being used to provide medical assistance and monitor health conditions. Wearable devices and apps can help track heart rate, blood pressure, and other important health metrics. They can also be used to alert medical personnel in case of an emergency. For instance, the Apple Watch Series 6 includes a feature that can detect if the user has suffered a hard fall and will alert emergency services if they are immobile for more than a minute.

Integrating AI technology into these gadgets may also give rise to new opportunities in regard to monitoring and tailoring IBS treatment. In 2022, Pimentel et al. published a study on this topic in the American Journal of Gastroenterology [40]. They developed a smartphone application using AI (named Dieta mobile app) for self-reporting stool form assessment. Out of the 45 IBS subjects included in the study, 39 agreed to use the application. Using digital images of users’ stools as input, the AI was trained to identify the characteristics of the stool. The visual characteristics used to train the AI were the Bristol Stool Scale, consistency, fragmentation, edge fuzziness and volume. The results of the study indicate that the AI system was significantly more accurate than the subjects’ own reports when categorizing daily average Bristol Stool Scale scores as constipation, normal, or diarrhea (0.95 vs. 0.89) [40].

In conclusion, AI may be able to provide a more objective assessment of stool characteristics, allowing for a more efficient diagnosis and treatment follow-up of IBS patients.

## 4. Future Trends

Although IBS has been a significant healthcare and economic problem for some time, little progress has been made regarding the paraclinical investigation of this disease. As new technologies are developed in medicine, some potential new tools may become available. To the best of our knowledge, there has only been one systematic review published on AI and IBS in 2022 by Marzieh Kordi et al. [41]. They were only able to include 20 papers in their research. Reviewing some of these papers revealed a more technical approach to IBS and AI than a clinical one [41]. Even though the authors concluded that AI algorithms can play an important role in predicting, diagnosing, and managing IBS, further studies involving close collaboration between physicians and computer experts are necessary.

As AI is becoming capable of performing more complex tasks with similar accuracy to human counterparts and a higher level of computing power, there is increasing potential to develop new tools that may aid with everyday tasks. The use of AI is becoming increasingly prevalent in many areas, particularly in the medical field. This will further improve the quality of not only the diagnostic process but also of individualized therapeutic strategies.

A few ideas for future AI developments in the management of IBS patients are presented below:

➢ Diagnose IBS early by analyzing patient data, symptoms and patterns, allowing for a more accurate and timely diagnosis.
➢ A personalized treatment plan can be developed by AI, based on a patient’s personal data, lifestyle and preferences, thereby optimizing symptom management.
➢ An AI-powered application can continuously monitor symptoms, providing real-time feedback and suggesting changes to diet or lifestyle.
➢ AI chatbots and virtual assistants can provide instant answers to IBS patients’ questions and assist them in managing their symptoms.
➢ By using Natural Language Processing (NLP) algorithms, it is possible to extract valuable insights from patients’ descriptions of their symptoms and experiences, which in turn can be used to assist in diagnosis and treatment.
➢ AI can provide personalized dietary advice, helping patients identify trigger foods and create IBS-friendly meal plans.
➢ Incorporating AI into telemedicine consulting can enhance the quality of telemedicine consultations by providing physicians with decision support and assisting them in making better treatment recommendations.
➢ By analyzing vast datasets and identifying potential therapeutic targets, AI can accelerate IBS treatment discovery.
➢ An AI-driven platform can facilitate the connection between patients with IBS and support communities and resources, fostering a sense of camaraderie and sharing coping strategies for IBS.

A particularly interesting approach for understanding IBS through the use of AI is to analyze the microbiome, which has been suggested to play an important role in IBS pathophysiology. Studies of microbiomes can be challenging due to the large number of different types of data available. AI can play an important role in processing these data at high speeds and establishing connections between the information presented. There have already been some studies in this field that have produced promising results. In one study, Hirokazu Fukui et al., 2020 investigated the relationship between IBS patients and the gut microbiota using Machine Learning-Based Microbiome Analysis. Using machine learning, they developed a prediction model for identifying IBS patients with a sensitivity of >80% and specificity of >90% [42].

In the future, it may be possible to use an AI system in order to evaluate an individual’s lifestyle, external factors, social interactions, as well as working and daily habits. Consequently, some potential causes of bowel disorders may be identified. Additionally, AI might also be able to provide personalized recommendations on dietary and therapeutic strategies. Integrating AI algorithms into portable gadgets such as smartphones or smartwatches could be a promising approach. Based on a continuous stream of data from daily life, the AI will potentially be able to recommend lifestyle changes tailored to the person’s individual circumstances.

## 5. Conclusions

Given its recent rapid advancement, it appears that technology development will keep on expanding in the years to come. Some concerns have been raised regarding the fact that humans are becoming increasingly dependent on machines and less able to perform certain activities without the use of these tools. However, it is essential to note that technology has enabled us to accomplish so much more in a fraction of the time, while at the same time maintaining the highest possible standard of quality in our activities. Undoubtedly, this is particularly prevalent in the field of medicine. Although this delicate balance between the dependence on machines and computers and our personal development must be carefully maintained, technology, including AI systems, can be greatly beneficial if properly used.

To sum up, IBS is unquestionably a rather challenging disease to diagnose and treat. Nevertheless, recent progress in AI development hints at the fact that many of our questions may be answered once a certain technological threshold is reached.

## Figures and Tables

**Table 1:** Currently approved colonoscopy computer-assisted tools for commercial use (modified after Taghiakbari et al. 2021) [13,23].

Product	Manufacturer	Place of Approval and Year	Computer System Used
EndoBRAIN	Cybernet System Corp./ Olympus Corp.	Japan 2018	CADx
EndoBRAIN-EYE	Cybernet System Corp./ Olympus Corp.	Japan 2020	CADe
EndoBrain-PLUS	Cybernet System Corp./ Olympus Corp.	Japan 2020	CADx
EndoBrain-UC	Cybernet System Corp./ Olympus Corp.	Japan 2020	CADx
GI Genius	Medtronic Corp.	Europe 2019 United States 2021	CADe
ENDO-AID	Olympus Corp.	Europe 2020	CADe
CAD EYE	Fujifilm Corp.	Europe 2020 Japan 2020	CADe/ CADx
DISCOVERY	Pentax Corp.	Europe 2020	CADe
WISE VISION	NEC Corp.	Europe 2021 Japan 2021	CADe
CADDIE	Odin Vision	Europe 2021	CADe
ME-APDS	Magentiq Eye	Europe 2021	CADe
EndoAngel	Wuhan EndoAngel Medical Technology Company	China 2020	CADe
